# Medical Students at Risk: Practical Lessons after 10 years of a Premedical Enrichment Program

**DOI:** 10.15694/mep.2019.000175.1

**Published:** 2019-09-17

**Authors:** Pamela Houghton DeVoe, Marcy Osgood, Nancy Shane

**Affiliations:** 1 University of New Mexico School of Medicine

**Keywords:** medical education, pre-medical education, student trust, preparation for medical school, post-baccalaureate programs, medical curriculum

## Abstract

This article was migrated. The article was marked as recommended.

**Introduction:** At the impending conclusion of a ten-year post baccalaureate program, the authors evaluated our Premedical Enrichment Program (PrEP) for effectiveness and component transferability to the medical curriculum. The study focused on two research questions: 1) was the PrEP successful in matriculating well prepared educationally disadvantaged students to medical school, and, 2) What did PrEP students value most about the program?

**Methods:** We matched 61 PrEP participants to a nonparticipating student of the same cohort, age, ethnic status, gender, economic disadvantage, MCAT score, and undergraduate GPA. We compared Step 1 performance and academic performance. An online survey tool collected student views on PrEP program effectiveness and retrospective pre-post questions regarding academic preparedness.

**Results:** PrEP student academic outcomes were equal to those of the matched comparison group despite higher MCAT scores and GPAs for the latter. Students valued the strong program relationships they built, ensuring study partners and emotional support during medical school. Students reported that the PrEP increased biochemistry knowledge, improved study skills, familiarized them with medical school processes, and introduced them to an active learning pedagogy. They expressed gratitude for having the opportunity to study medicine. They felt the program built their confidence, encouraged professional identity exploration, and that their diverse experiences and challenges were valued.

**Discussion/Conclusion:** Results reiterate the importance of the social learning environment, the potential to strengthen academic performance by building genuine and long lasting relationships, and the subsequent potential for growth in confidence for medical school. Interconnected course content reinforces new learning and student confidence with learning strategies. Important goals for educationally disadvantaged student success include building these trust relationships with significant faculty and student colleagues. Confidence and trust have the opportunity to grow with authentic performance successes, witnessed, shared, and confirmed by sympathetic peers and faculty.

## Introduction

Training sufficient physicians to address the needs of healthcare shortage areas is an ongoing concern for many US states and globally. The University of New Mexico, School of Medicine (UNM SOM) has a mission, in part, “to advance the health of all New Mexicans by educating and increasing the diversity of health professionals, leaders and scientists” (UNMSOM, 2018). A successful avenue for increasing the number and diversity of practicing physicians has been the SOM post-baccalaureate program, designed to improve educationally disadvantaged students’ readiness for the medical curriculum. This paper describes lessons learned after 10 years of the Premedical Enrichment Program (PrEP) at UNM SOM. We present these lessons in terms of institutional context, student performance, and student reflections.

### Background

#### Premedical Enrichment Program (PrEP) Development and Overview

In 2007, a group of UNM medical school faculty and educational staff designed a unique post-baccalaureate pre-medical curriculum. We considered our institutional experience plus other schools that built post-baccalaureate enrichment programs for educationally disadvantaged students ( Giordani *et al.*, 2001; Lipscomb *et al*., 2009; Blakely and Broussard, 2003; Grumbach and Chen, 2006). As educators, we used our familiarity with instructional and learning theory literature to incorporate best practices. We wanted to guide students toward learning self-regulation as well as self-confidence; we wanted them to acquire medical science knowledge as well as the best ways to master that knowledge. Selection criteria included undergraduate overall and science GPA; MCAT score; non-cognitive skills such as professionalism, initiative, resilience, and commitment to community; New Mexico residency; and a (self-reported) educationally disadvantaged background. We followed the U.S. Department of Education Office of Postsecondary Education definition (34 CFR 606.7), which includes, but is not limited to “students who come from: 1) economically disadvantaged families; 2) limited English proficiency families; 3) migrant worker families; or 4) families in which one or both parents have dropped out of secondary school.” The SOM Admissions Committee recommended students from the general medical school applicant cohort, and PrEP faculty selected students based on further review.

The PrEP curriculum varied slightly over its 10-year history as faculty availability changed, but PrEP was always a “bespoke” program, designed deliberately for our students. PrEP program goals were to facilitate the development of the knowledge, skills, and attitudes needed by educationally disadvantaged students to be successful in medical school and eventually to become providers of high-quality healthcare to our communities. To achieve these goals we drew upon two influential theoretical frameworks; social constructivism, which highlights the role of learners’ group(s) in the learning process ( Bodner, Klobuchar, and Geelan, 2001) and cognitive apprenticeship, which uses expert modeling and discussion to help students develop confidence, critical thinking and self-assessment skills ( Collins, Brown and Holum, 1991; Stalmeijer, 2015). Elements of these frameworks coalesced into our program’s main facets:

• Small learning groups-- (seven students, one instructor) to promote students’ feelings of comfort, safety, and interdependence;
• Meaning construction--students were guided to construct meaning with new content by connecting new information with that already known, to reflect, assess, and complete their understanding by incorporating ideas from group discussions;
• Self-regulated learning--to move students from their somewhat passive undergraduate learning orientation toward a more self-determined, active learning platform, which would be required in medical school ( Nietfeld and Schraw, 2002; Zimmerman and Schunk, 2001);
• Mentoring and modeling of professional cognitive behaviors--to build a learning environment that facilitated faculty and student mentoring relationships; and where faculty (simultaneously involved with the medical school) could demonstrate learning process as well as ease with the medical curriculum;
• Learning and organization practice--students were encouraged to practice newly taught learning strategies and organizational study skills within the PrEP courses to develop flexibility in anticipation of the medical school learning environment.

Review of courses. The PrEP curriculum followed the regular non-medical campus calendar with two 16-week semesters. In implementation the general curricular objectives were to provide students: 1) a solid foundation in the sciences basic to medicine; 2) enhanced critical-thinking and problem-solving abilities; 3) individualized development in lifelong learning skills; 4) improved verbal and written communication skills; 5) an introduction to community medical practice; and importantly, 6) improved confidence and peer support for success in medical school. The PrEP curriculum had several component courses:

*Biochemistry.* The two-semester Intensive Biochemistry sequence for undergraduate biochemistry majors, completed with a B- or better in both courses. The biochemistry classes integrated a variety of active learning opportunities and assessment modalities. Biochemistry was the central basic science course based on the intrinsic integrative nature of the discipline and on learner problem-solving strategy research of undergraduate Biochemistry students by one of the authors (MO) ( Anderson *et al*., 2011; Sensibaugh *et al*., 2017).

*Reflective Writing.* Students wrote both in and outside of class, kept a notebook of observations, and responded to books and short pieces (fiction, non-fiction, drama, and poetry) about various aspects of medical practice and general life experience. The PrEP students shared their writing, and thereby learned in depth about each other.

*Lifelong Learning.* This course directed students on ways to improve their study strategies, test taking skills, organizational skills and professionalism. Designed by one of the authors (PD), an Educational Psychologist and director of the medical school’s academic support office, instructional emphasis was on self-directed learning, growth mindset, and study skills flexibility. A SOM learning specialist also facilitated wellness activities that students instructed.

Society and Medicine. An introduction to public health, social determinants, and cultural and societal concerns in the access to, and provision of healthcare. Students read and discussed relevant research, completed research papers, and participated in fieldtrips. A one-semester community-based service-learning component allowed students to shadow physicians in a primary care clinic, reflect on the experience, and create a project of relevance to clinic operations.

*Medical Science Foundations and Correlations.* Problem Based Learning (PBL) and Case-based Learning (CBL) modalities introduced students to human body systems, normal and pathophysiological. The goal was to develop critical-thinking and problem-solving skills, and help students master both the basic science content and learning processes. Faculty designed cases to integrate with the biochemistry, and introduced other sciences basic to medicine such as anatomy and physiology.

### Purpose of the study

Periodic evaluations throughout the program’s ten years made it clear that PrEP students performed on par with directly admitted students in almost every medical school class. As we prepare to conclude the program due to changing personnel availability, we designed a study to capture student views on the most valuable aspects of the program as well as medical school performance outcomes.

This study focused on two research questions:

1. Was the PrEP successful in matriculating well-prepared educationally disadvantaged students to medical school?

2. What did PrEP students value most about the program?

## Methods

We examine outcomes and feedback from the ten cohorts of students who have completed the PrEP curriculum and matriculated to the UNM SOM. Compared to directly admitted students, PrEP students were on average older, more often from an ethnic or racial group that is underrepresented in medicine (UIM), more likely to be female, more likely to be economically disadvantaged, and more likely to come from a rural area of the state. (See Appendix A) We matched each of the 61 consenting PrEP participants to a nonparticipating student of their same cohort, as similar as possible in age, ethnic minority status, gender, economic disadvantage, MCAT score, and undergraduate GPA. The comparison group had no statistically significant demographic differences with PrEP students; however, they did have statistically significantly higher MCAT scores and undergraduate GPAs. (See Appendix A) Thus, to answer the first research question, we compared the PrEP students and similar peers in Step 1 performance and academic progress, using Student *t*-tests.

To answer the second research question, we used “Survey Monkey” an online survey tool ( Appendix B), successfully reaching 52 students from ten cohorts of PrEP participants from academic years 2007-08 to 2016-17. The participants now range from first year medical students to post-residency physicians. We did not send surveys to participants who had left medical school. We designed the survey to gather student feedback on the PrEP curriculum components, using Likert-type questions to rate program effectiveness and retrospective pre-post questions to examine student views on preparation for medical school. We received 24 responses for a response rate of 46%. We used the same online tool to collect 17 “Most Significant Change” (MSC) style narrative stories, a response rate of 33%, to gather rich and contextualized feedback ( Davies and Dart, 2003). Of the 17 stories, 16 were positive in tone, describing one or more significant changes; and one was a negative “challenge” story, suggesting no significant change during PrEP. In this mixed-method design, we used conventional content analysis to analyze the MSC stories to identify themes, weighing their importance according to prevalence and concordance with the descriptive statistics results garnered from the quantitative survey questions Hsieh and Shannon, 2005).

This study was approved by the University of New Mexico Health Sciences Center, Human Research Protections Office, September 22, 2017, protocol # 17-336.

## Results/Analysis

### Research Question #1. Was the PrEP successful in matriculating well-prepared educationally disadvantaged students to medical school?

Despite statistically significantly lower pre-matriculation academic metrics (MCAT scores and college GPA), PrEP students at UNM have progressed through medical school successfully and are statistically just as likely to graduate as their similar peers are. Among the seven cohorts for whom graduation has been possible, 81% of PrEP students and 88% of comparison students graduated (statistically equivalent, p=.90). As seen in Table 1, among all ten cohorts, 66% of PrEP students remain/remained on-schedule compared to 72% of their similar peers. This difference is not statistically significant; in fact, most of the difference comes from the PrEP students taking more personal leave than similar peers do. There is almost no difference in the students who delayed due to an academic upset; and no percent difference in the number of students who were dismissed or withdrew from medical school.

**Table 1.** Academic Progress: PrEP Students and Similar Peers Remain On-Schedule at the Same Rate. UNM SOM, 2017-2018.

	PrEP	Similar Peers	*p*	*t*
Total Matriculants, 2008-2017	61	61		
Graduated/Enrolled: On Schedule *	40/66%	44/72%	.44	.78
Graduated/Enrolled:Off Schedule - Personal Delay	4/7%	1/2%	.17	1.37
Graduated/Enrolled: Off Schedule - Academic Delay	15/25%	14/23%	.83	.21
Dismissed	2/3%	2/3%		

* Note: “On Schedule” includes students who take more than four years to graduate due to PhD or other advanced study programs.

A key outcome during medical school is USMLE Step 1 passage and score. As indicated in Table 2, below, despite lower incoming MCAT scores for PrEP students compared to similar peers, average Step 1 scores are nearly equivalent, and the difference between groups is not statistically significant. The results suggest that in ten years of the program, the PrEP participants have succeeded as well as their similar peers.

**Table 2.** PrEP students and Similar Peers Perform Equivalently on Step 1. UNM SOM, 2017-2018.

	PrEP	Similar Peers	*p*	*t*
Total Matriculants, 2008-2015	49	49		
Have Taken Step 1	46/ 94%	47/ 96%	.65	.46
Passed Step 1, 1 ^st^ Attempt	72%	85%	.12	1.57
Average Score, 1 ^st^ Attempt	204.0	205.5	.73	.35
Range of Scores	161 - 252	156 - 242		

### Research Question #2: What did PrEP students value most about the program?

**Figure 1.**
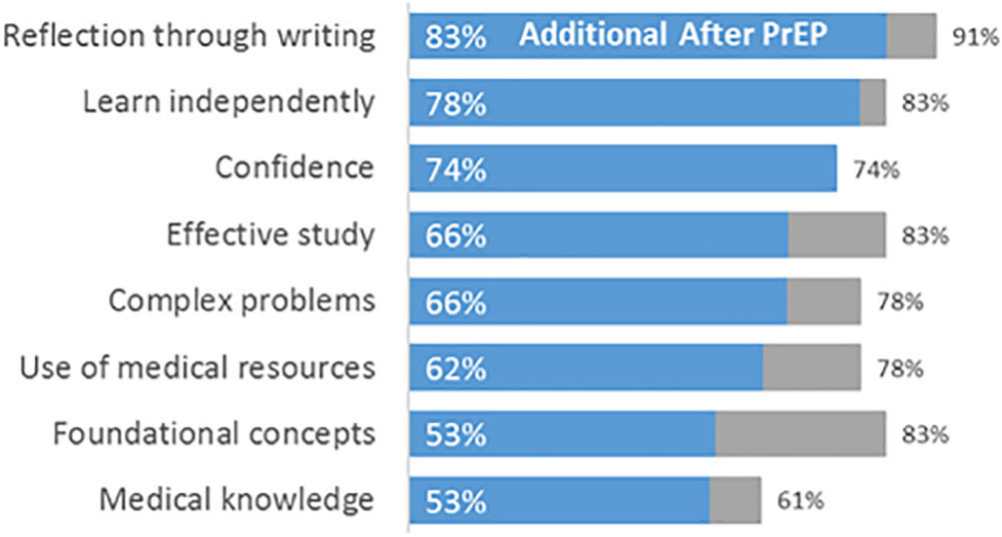
Change in Percentage of Students Feeling Prepared for Medical School

We asked students their perspectives on which components of the PrEP they found valuable. First, students rated their level of preparation (0-4 scale) for a number of skills before PrEP, and at the beginning of medical school, after PrEP. Figure 1 shows, at the far right of each bar, the total percentage of students indicating they felt “highly” or “well” prepared after PrEP, (3-4 on the scale). The percentage indicated on the left-hand side of the bars as well as the length of the bar indicates the percentage of such students who also reported that they had not felt well- or highly- prepared before PrEP - thus, the amount of positive change.

**Figure 2.**
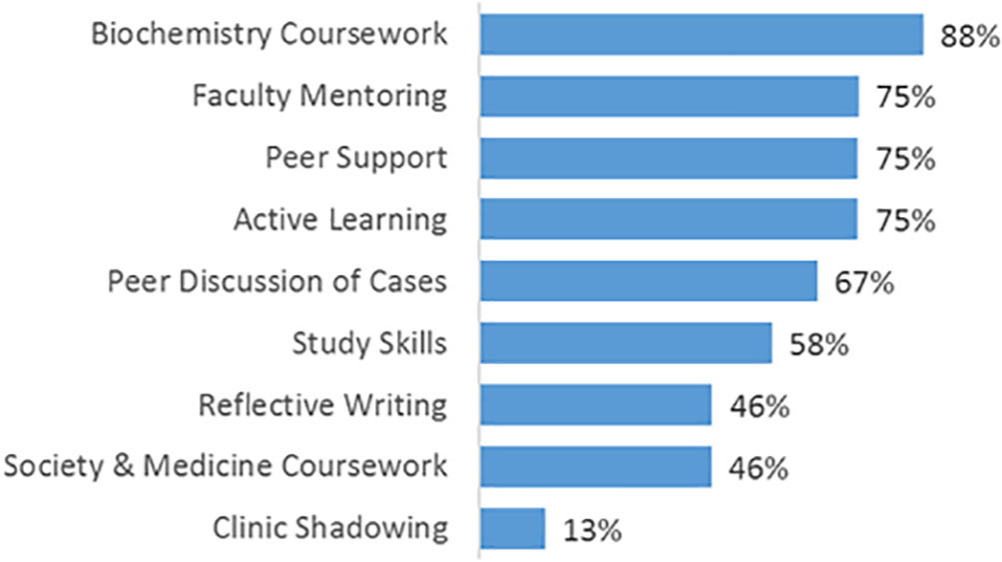
Percentage of Students Reporting the PrEP Component as “Highly Effective” in Preparing Them for Medical School

Second, as seen in Figure 2, participants rated the effectiveness of program components or activities in preparing them for medical school on a 0 (“Not very effective”) to 2 (“Highly effective”) scale. See the complete survey tool in Appendix B.

Regarding the qualitative data, Table 3 lists salient themes identified in students’ MSC stories. We grouped these themes into three content areas: 1) the PrEP “Ethos”; 2) components of PrEP that students thought contributed to their academic success; and 3) long-term outcomes. While the prevalence of an idea should not be overrepresented in qualitative research, we do list the themes within each content area from most to least common; and we organize the rest of the Results section in the same way. We describe each theme using both student quotations and applicable quantitative survey information from Figures 1 and 2.

**Table 3.** Salient Themes in Students’ Most Significant Change Stories. UNM SOM, 2017-2018.

PrEP Ethos
Opportunity to Study Medicine
Building Confidence
Personal Reflection and Sharing
Respecting Students’ Personal Journeys
**Attributions to Academic Success**
Relationship with other PrEP Students
Biochemistry Coursework
Faculty Mentoring
Group Work
Study Skill Instruction
Familiarity with the Place and Process
**Long term Outcomes**
Life-Long Relationships
Professional Aspirations

### The PrEP Ethos

Opportunity to Study Medicine. Several student MSC story titles such as “Fortifying My Dream with a Second Chance” and “The Chance” implied that the authors understood their lower academic scores put them at risk for denial of admission to medical school. In their stories, over four in five students expressed gratefulness for the opportunity, sometimes perceived as their last chance, to attend medical school. Many expressed becoming a physician as their dream. We list representative student quotes below.

• The most significant result of PrEP is realizing my dream of becoming a physician. PrEP gave me so much starting with the chance to prove myself in the program and in medical school. It is because of the program that I succeeded, and am realizing my dream of practicing medicine presently as a resident physician in New Mexico.
• PrEP was the only way I could have gotten into medical school. My G.P.A. was under the requirement for the school of medicine and I had so many credits from my undergraduate degree that even getting A’s in numerous graduate courses was not improving my G.P.A.
• Although I was likely seen as a risky academic gamble by the admissions committee I would like to think I have demonstrated the benefits of taking students with unique backgrounds, experience, and demonstrated educational “Grit,” giving them the tools to succeed and seeing what we are capable of.
• There is a real need for opportunities for students who are educationally disadvantaged. The fact that the program looked at multiple different minority statuses was vital.

Building Confidence. When asked about their level of confidence to be successful in medical school before PrEP, no student rated his or her level above 2 (“moderately confident”) on the 0-4 scale. Almost 3 in 4 (74%) reported they were “extremely” (4) or “very” (3) confident by the beginning of medical school (after PrEP). (See Figure 1).

• The PrEP gave me my confidence but also helped me create the bonds that I rely on still to help me in medical school.
• Prior to joining PrEP, I had applied for medical school twice and had since graduated from undergraduate school for several years. PrEP gave me the tools and confidence I needed to make sure I would excel in medical school.
• What I believe I got the most out of the PrEP program is the confidence to be successful in medical school. The bonds I made with my colleagues in PrEP gave me a strong support system for medical school and also lifelong friends.
• If not for PrEP, I would not be the confident and happy medical student that I am today.

Personal Reflection and Sharing. Personal reflection proved a major tool for student introspection and development of metacognitive and stress management skills. The Reflective Writing course was the main avenue for exploring personal beliefs, values, and interpersonal understanding. Interestingly, when asked about the effectiveness of reflective writing, fewer than half rated it as “highly effective” ( Figure 2). On the other hand, more students reported significant improvement in this skill compared to all others ( Figure 1). The great majority, 91%, felt “highly” or “well” prepared in this skill by the beginning of medical school. Several students suggested Reflective Writing helped them to learn about and bond with other PrEP students; and they described its role in building professional identity.

• I first interacted with the people from PrEP because it was required [in] the curriculum (Reflective Writing), we were exposed to each other’s thoughts, feelings, and stories. I learned that I was not that different from other people in my feelings. We all came from different backgrounds but we all had a common goal and this brought us all together.
• And while I didn’t appreciate it much at the time, the writing portion has had long lasting effects on me and is a tool I sometimes use now for coping with the stress that is inevitable in medicine.

Respecting Students’ Personal Journeys. The concept of educational disadvantage often includes personal, language, familial, medical, social, and financial challenges that students have had to overcome. Many students appreciated that the challenges they faced in their lives were not counted against them, but rather seen as experiences that made them stronger and more resilient.

• PrEP looked at me as a person and took time to get to know me and my story, instead of seeing me as just numbers on a paper. They gave me a chance at an interview and took my whole life into account and did not compare me to people who had very different backgrounds from me. They saw the progression and the recent improvement in my academic performance as well as recognized my resilience and other strengths.
• Along my journey to medical school, there were several bumps in the road, personal, social, and other, that could have been seen as demerits on my medical school application. The Admissions Committee was able to see these circumstances and count them as merit, rather than demerit, and evidence of a life lived and learned from.
• Growing up in a single-parent household, I always felt as though .. I could never attain my dreams. I entered college as a homeless student, working two jobs to be able to pay for school and save enough money for a place to live. Even then I never imagined being accepted to and being able to attend medical school. I finished my undergraduate, soon applied to medical school and during my interview .. I was told about the PrEP and about how I could apply for it.
• The truth of the matter is many of us have stories. We come from disadvantaged backgrounds, but sometimes our backgrounds don’t quite fit into the checkboxes of medical school applications. PrEP values my history and my diversity and, for the first time, I really felt like maybe my life experiences had value and were important to my future identity as a medical student.

### Attributions to Academic Success

Because the ultimate curricular goal for the PrEP designers was to prepare students to succeed in medical school and to graduate, it was critical to help students hone their academic preparedness as much as possible. Of the 17 MSC stories, 16 described or implied academic preparation as an important benefit, generally defining academic “success” as graduation from medical school. See relevant quotes immediately below. Though quotes in the following sections emphasize how specific PrEP components contributed to students’ success, we understand that many students did not ascribe discrete PrEP components as crucial to helping them succeed but, rather, understood the components as working together as a greater whole.

• Through the program, I worked hard and felt better prepared to start medical school. It was not just the biochemistry course work but also the reflections, the working as a team with fellow PrEP students/friends that made me who I became, someone actually ready to take on the longer journey that lay ahead.
• Once PrEP gave me the opportunity to enter medical school, (it) also gave me education and skills to better prepare myself..I went through an intense biochemistry curriculum ..that strengthened and sharpened foundational science for me. More importantly, (it) helped me develop group and individual study skills that I could carry with me into medical school.

Relationships with other PrEP Students. Seventy-five percent of students rated Peer Support as highly effective, and 67% rated Peer Discussion of cases as highly effective (See Figure 2). The following quotations show that students relied on each other in various ways.

• The team mentality grown through the program is one of its intangible benefits. The cohort that I went through the program with is my core support group in medical school and the guidance offered by previous cohorts is something that gives me an advantage in medical school.
• Lastly, one of the biggest gains I had from PrEP was gaining a close network of peers and faculty that became a medical school family/support system. I had people who knew me very well, including my strengths and weaknesses. It was a small, intimate environment and we had the opportunity to teach each other about where we came from, who we were, and what we valued.
• .. Furthermore, this experience established relationships between a diverse group of individuals that have varying backgrounds, cultures, and beliefs, which.. was so impactful on all of our lives.
• When I felt discouraged, I had people who understood what I was going through who could pick me up. I had those same people who could look to me when they needed the same. I had people I could check in with regarding assignments or exams, people who I knew I could trust.. Having this type of support system in medical school is astounding. It can be difficult sometimes to look for this type of support from family and other friends, because as much as they try, they just cannot fully relate or understand. PrEP gave me that.

Biochemistry Coursework. Fifty-three percent of students felt more prepared with foundational scientific concepts after PrEP, and 88% rated the biochemistry coursework as highly effective. (See Figures 1 and 2.)

• The biochemistry exposure was essential and provided a strong foundation to many concepts that emerged during medical school.
• (Biochemistry).. at least for the first two years of medical school, (was) the most beneficial course.
• The PrEP really gave me a taste for what was to come. The way the biochemistry course was set up was so perfect. It mimicked the way the medical school classes would be taught.

Faculty Mentoring. Seventy-five percent of students rated faculty mentoring as highly effective. (See Figure 2.)

• PrEP provided exposure to faculty, many of whom have continued to support me throughout my career, provided exposure to resources, opportunities for support such that I was able to start medical school with both feet on the ground, having an already built-in support system that allowed me to focus solely on my studies.
• I learned the value of professor office hours and how willing faculty (were) to help us if we needed any further clarification or education.
• The faculty also worked with us very intimately and all created a very approachable and supportive environment.

Group Work. (Problem Based Learning [PBL] and Case Based Learning [CBL]). Seventy-five percent rated active learning (in small group activities) as highly effective. (See Figure 2.)

• ..the importance of small group discussion for studying. Teaching each other the concepts (was) invaluable to me.
• We PrEP students worked together to study course material and we learned the benefits of group studying and quizzing. I continued to use these skills into medical school and could perform well..

Study Skills Instruction. Seventy-eight percent of students were more able to learn independently after PrEP; 66% of students were more able to study effectively; 66% of students were better able to solve complex problems; 62% of students were better able to use medical resources; 58% of students rated study skills instruction as highly effective. (See Figures 1 and 2.)

• More importantly, (it) helped me develop group and individual study skills that I could carry with me into medical school. PrEP introduced me to table based learning (small group learning where we work together to solve a case and teach each other learning issues). I realized that teaching information to my peers using only a whiteboard pushed me to truly understand the material myself.
• PrEP gave me the tools to think about the best ways to study for different curricula, switching from concept maps to tables or charts and sequential learning. I learned to quickly adapt my study techniques to best suit the task at hand and because of this I acquired knowledge in a way that through 3rd and 4th year has directly benefited my patients and my team.
• I garnered a skill set during PrEP that has proven to be successful not only in PrEP, but also in medical school. Additionally, it is so empowering to know that I can feel confident in the way that I am studying, and know that I will do well in medical school.

Familiarity with the Place and Process. Some MSC stories highlighted the benefit of an early introduction to the medical school.

• Feeling comfortable with the expectations and demands from medical school was one of the most valuable parts of PrEP for me.
• Having the familiarity with the campus, the health science library, and many faculty in the school of medicine was seriously helpful. When I showed up on the first day of medical school, I knew exactly where I was going and who I was going to sit with. I then knew exactly where I planned to study or do group work. I also knew what resources were available in the library and how to access them. I feel like this instantly set me apart from my classmates.
• PrEP made me become familiar with the city.., campus, and other resources so I did not feel totally alone when medical school started.

### Long-term Outcomes

Life-Long Relationships. A few participants describe relationships and friendships that extend beyond medical school and have a depth that feels life-long to them.

• Technically speaking, the biochemistry was very helpful, but [a] huge part of medical school is working through the day-to-day rigors, staying engaged and trying to enjoy the little things as you pour ALL of yourself into your studies. PrEP provided me the opportunity to make friendships that I suspect will last for many, many years. Friendships forged in the fire are likely to last forever, and I feel that PrEP gave this to me.
• The most significant result of PrEP is the amazing support system that came with it. The 5 amazing friends that have made my medical school experience one that I could bear..We have provided each other with encouragement when things got difficult, attended each other’s’ weddings, celebrated new children, and picked each other up during serious trials.. I know that I will have these friendships for a lifetime, and that one day when we are all celebrating our retirements together, we can all look back and thank the PrEP [for] making it all possible.
• I was part of PrEP in 2007. I cannot recall specific details about my time during PrEP.. What I do remember is the lasting relationships that I made during that time.. All I can really say is that the friendships I made during PrEP (with my fellow preppies) have significantly contributed to making me the physician that I am today.

Professional Aspirations. An unexpected long-term outcome identified by some PrEP students was their expanded professional aspirations, such as academic medicine. Others described PrEP as contributing to their altruism, professionalism, and leadership.

• Simply put, PrEP has not only given me the opportunity to attend medical school, it gave me the tools to thrive as a student leader. While participating in PrEP I learned better study habits and gained introspective techniques and metacognitive tools..As a (medical) student I had the privilege to help tutor and teach other classmates and students using many of the techniques and habits I developed working with [PrEP faculty and staff]..I have a strong ambition to pursue an academic medicine career and hope to someday participate as an instructor or facilitator for the PrEP program.
• ..I believe without a shadow of a doubt that the students matriculated through the PrEP program go above and beyond to fulfill their duty and commitment to the mission of UNM SOM, and this is evidenced by the high number of PrEP graduates currently serving the underserved throughout New Mexico and teaching at UNMSOM.
• [I] feel as if PrEP enabled me to be successful in various avenues of my life. The academic advantage PrEP provided for my cohorts and I is obvious, but I also feel strongly that PrEP cultivated our sense of introspection, wellness, and awareness of social determinants of health, altruism, leadership ability, networking capacity, and professionalism.

PrEP students give back to the SOM in substantial ways. Two graduates from the first cohort are now SOM faculty members; several PrEP students served on the Curriculum Committee, the Admissions Committee, or the Committee for Student Promotion and Evaluation (CSPE). Two former PrEP students created a sustainable peer-tutoring effort. Residency and practice patterns also reflect the PrEP ethos; PrEP graduates are somewhat more likely to elect to stay in New Mexico for residency training (53%) compared to their similar peers (45%), and much more likely than all other SOM students (29%) are.

## Discussion

### Practical Lessons: Translatable Elements from the PrEP to Premedical and Medical Curricula

PrEP was successful in matriculating well-prepared educationally disadvantaged students to medical school. PrEP students’ academic outcomes were equal to those of the matched comparison group despite higher MCAT scores and GPAs for the latter. Study results reiterated for us the importance of the social learning environment, possibly one of the PrEP’s most transformative aspects. Students in this study expressed great appreciation for personal reflection and sharing, faculty mentoring, and, especially, building relationships with other students. Students and faculty cohesion developed with each cohort of PrEP students based on shared goals and mutual respect. Students describe both the forming of “bonds” with fellow PrEP students, as well as an increased confidence for medical school. Students learned to trust each other as study partners, as fellow students with similar backgrounds, and many developed genuine long-lasting friendships. PrEP’s small group learning environment and open, safe, and sensitive discussions seemed to create an atmosphere conducive to trust building. These trust relationships grew from regular, supportive academic interactions and not from social interactions, as is sometimes the model.

We acknowledge the difficulty of translating components from a small program to a large medical school population. We suggest, however, the possibility that similar quality relationships might emerge in medical school with the right encouragement and learning environment. One possible example is the relatively recent development of Learning Communities, composed of small groups of medical students and one clinical faculty mentor. These dedicated groups, intact over the four years of training, show promise in simulating this trust relationship, and have successfully contributed to the student-teacher relationship in participating medical schools ( Ferguson *et al*., 2009). One recent study found that trust was a stronger predictor of student outcomes than even student “growth mindset,” highlighting the importance of building student trust as a means for improving learning outcomes ( Elks *et al*., 2018; Cavanaugh *et al*., 2018; Dweck, 2008).

Another potentially translatable PrEP aspect is the contextual learning how to learn that was possible due to the small class size and cohort course schedule. Study participants appreciated the specific study skills instruction they received. Guidance on learning strategies and flexibility in their use makes more sense to students when they can apply the strategies to their current course content. This guided practice, especially with relatively low stakes exams, is potentially more motivating than “stand alone” presentations or written materials.

Many medical schools and other health professions training programs are currently struggling with introducing more student-centered and active-learning modalities into their curriculum, and experiencing push back from both students and instructors (Cavanaugh *et al.*, 2016; White *et al.*, 2014; Bossaer *et al*., 2016; Persky and Dupuis, 2014). Because we introduced PrEP students to active learning in both a large class (Biochemistry) and in small groups (all other PrEP courses) in the year preceding medical school, they were comfortable and prepared for the expectations of such pedagogies. Indeed, participants suggested group work to be an important component to their academic success. Buy-in is one of multiple curricular aspects that affect student performance in active-learning courses ( Yengo-Kahn, Baker and Lomis, 2017). We propose that actively building a learning environment and climate of faculty-student trust may contribute to pedagogical and student academic success.

Some of the appreciated components of PrEP are unique to small settings; so this study also has lessons particular to small group premedical curricula. One of the major themes of the MSC stories was explicit appreciation for having the opportunity to attend medical school. Premedical programs tend to accept *a priori* highly motivated, hardworking students-a definite ‘plus.’ As mentioned, PrEP benefitted from its intentional practices of building confidence, in part through respecting students’ unique and often challenging journeys. Finally, programs should tailor their curricula to the students they have. For example, the clinical shadowing component of PrEP was rarely mentioned in our survey, possibly because many PrEP students had already had such experiences. Similarly, PrEP students appreciated the efforts to familiarize them with campus more than expected; many had been away from an academic setting for some time.

At the beginning of medical school, most PrEP students were noticeable for their composure, eagerness, and confidence. Building confidence to succeed in medical school for educationally disadvantaged students was an important goal for PrEP. Confidence grew from authentic performance successes, and were witnessed, shared, and confirmed by sympathetic peers and faculty. Medical schools could revisit the paradigm of targeting opportunities for building confidence in their students, as well as providing opportunities to master medical science content.

### Limitations of the Study

We used a self-report survey, including a retrospective pre/posttest, which is susceptible to social desirability bias ( Hill and Betz, 2005); our findings may indicate more favorable student perception about PrEP than is valid. Similarly, while our survey and MSC story response rates are acceptable (46% and 33% respectively) we collected a relatively small number of surveys (24) and stories (17). If the most enthusiastic students were more likely to respond, it could bias our findings. We also collected more surveys (62%) and stories (71%) from the four cohorts of current medical students than the six cohorts of graduated students. However, we found no discernable pattern or statistically significant differences in any quantitative measure by cohort status (graduated or current). In addition, PrEP was a highly tailored pre-matriculation program. We cannot necessarily generalize these results to other pre-matriculation or post-baccalaureate programs.

## Conclusion

Results reiterate the importance of the social learning environment, the potential to strengthen academic performance by building genuine and long lasting relationships, and the subsequent potential for growth in confidence for medical school. Interconnected course content reinforces new learning and student confidence with learning strategies. Important goals for educationally disadvantaged student success include building these trust relationships with significant faculty and student colleagues. Confidence and trust have the opportunity to grow with authentic performance successes, witnessed, shared, and confirmed by sympathetic peers and faculty.

## Take Home Messages

• Small size in post-baccalaureate programs may facilitate group cohesion between students and faculty.
• Students develop confidence in their academic and professional abilities through incremental, authentic successes.
• Students gain self-efficacy for medical training by proving themselves with difficult medical curriculum-oriented courses prior to medical school.
• Guidance from trusted faculty advisors and program peers prior to medical school can facilitate the transition into medical training.

## Notes On Contributors

Pamela Houghton DeVoe, PhD, Educational Psychologist, Learning Specialist, and Director, Office of Academic Resources & Support, Undergraduate Medical Education, University of New Mexico School of Medicine.

Marcy Osgood, PhD, Biochemistry Professor Emerita, Department of Biochemistry, University of New Mexico School of Medicine.

Nancy Shane, PhD, Program Evaluator, Office of Program Evaluation, Education, and Research, Undergraduate Medical Education, University of New Mexico School of Medicine.

## Appendices

### Appendix A: Description of PREP Students and a Comparison Group of Similar Peers

**Table:**

Total Matriculants, 2008-2020	PREP	Similar Peers ^2^	t	All other Students
*n*	61	61		727
Age	27.1	26.1	1.69	24.6
Female	59%	57%	0.18	53%
Under-represented Minority	76%	75%	0.11	42%
*Hispanic*	*55%*	*47%*	*0.91*	*32%*
*Native American*	*10%*	*18%*	*1.24*	*6%*
*African American*	*10%*	*12%*	*0.23*	*2%*
*Vietnamese*	*2%*	*0%*	*1.00*	*2%*
Rural?				
Economic Disadvantage (n=50)	80%	68%	0.96	25% ^3^
Overall College GPA	3.36	3.52	2.92 **	3.66
College Science GPA	3.13	3.32	3.56 **	3.57
MCAT ^1^	496.6	498.1	2.55 *	504.8

^1^ Old MCAT scores were converted: http://www.prospectivedoctor.com/mcat-score-converter/

^2^ Excludes BA/MD students, other Post Baccalaureate program participants, & transfer students

^3^ n=256

* p<.05, **p<.01

### Appendix B: Survey Questions

1. Consent.
2. Narrative. First, we would like to hear your reflections about the most significant result or aspect of PrEP for you. If you have already written your story (recommended), you may paste it from its original document. Please do not use students’ real names, including your own. There is no minimum or maximum length. Most participants’ stories consist of 4-6 paragraphs, with an additional 1 or 2 paragraphs describing its significance.
  a. What is the name of your story?
  b. Please tell the story in the space below (provided in on-line survey).
  c. Why is this story significant for you?
3. Survey. Thinking only of your personal experience, please provide responses for each statement below indicating your level of preparation for the skill at two points in time: first, BEFORE PrEP, and then, AT THE BEGINNING OF MEDICAL SCHOOL. Please fill the circle indicating your answer using the following categories: Highly prepared; Well prepared; Moderately prepared; Slightly prepared; Not at all prepared.

Survey questions are listed below:

4. Understanding scientific concepts foundational to medicine.
5. Knowing how to approach complex problems in order to make a decision or choice.
6. Ability to learn independently about medical topics of interest.
7. Ability to reflect on medical issues through writing.
8. Knowledge about medical practice.
9. Ability to study medical concepts effectively.
10. Ability to locate and use scientific and medical resources effectively.
11. Confidence to be successful in medical school.
12. How would you rate the following activities or aspects of PrEP in terms of preparing you for medical school? (We acknowledge there is some overlap. Please answer as best you can.) Please fill the circle indicating your answer using the following categories: Highly effective; Somewhat effective; Not very effective; Not applicable.

Content of biochemistry coursework

Active learning/Small group activities

Reflective writing assignments

Society & Medicine coursework

Clinic shadowing experience

Peer discussion of cases

Study skills & time management education

Mentoring by PrEP faculty

Student support of one another

13. Is there anything you would like to add about how well PrEP prepared students for medical school?
14. Finally, is there anything else you would like to add about your experience in PrEP that you have not yet had a chance to say?

## References

[ref1] Anderson W. Sensibaugh C. Osgood M. Mitchell S. (2011). What really matters: assessing individual problem-solving performance in the context of biological science. IJSOTL.(1). 10.20429/ijsotl.2011.050117

[ref2] Blakely A. W. Broussard L.G. , (2003). Blueprint for establishing an effective post-baccalaureate medical school pre-entry program for educational disadvantaged students. ACAD MED. 78(5):437–447. 10.1097/00001888-200305000-00004 12742777

[ref3] Bodner G. Klobuchar M. Geelan D. (2001). The many forms of constructivism. J CHEM EDUC. 78:1107. 10.1021/ed078p1107.4

[ref4] Bossaer J. Panus P. Stewart D. Hagemeier N. George J. (2016). Student Performance in a Pharmacotherapy Oncology Module Before and After Flipping the Classroom. AM J PHARM EDU. 80(2) Article 31. 10.5688/ajpe80231 PMC4827582 27073284

[ref5] Cavanagh A. Aragón O. Chen X. Couch B. Durham M. (2016). Student Buy-In to Active Learning in a College Science Course. CBE LIFE SCI EDUC. 15(4). 10.1187/cbe.16-07-0212 PMC5132373 27909026

[ref6] Cavanagh A. Chen X. Bathgate M. Frederick J. Hanauer D. (2018). Trust, Growth Mindset, and Student Commitment to Active Learning in a College Science Course. CBE LIFE SCI EDUC. 1–8.18. 10.1187/cbe.17-06-0107 PMC6007784 29378750

[ref7] Collins A. Brown J. S. Holum A. (1991). Cognitive apprenticeship: Making thinking visible. AM EDUC. 6(11):38–46. http://citeseerx.ist.psu.edu/viewdoc/download?doi=10.1.1.124.8616&rep=rep1&type=pdf( Accessed: 19 April 2019)

[ref8] Davies R. and Dart J. (2003). A dialogical, story-based evaluation tool: The most significant change technique. AM J EVAL. 24(2):137–155. 10.1016/s1098-2140(03)00024-9

[ref9] Dweck C. S. (2008). Mindset: The new psychology of success. New York: Ballantine Books.

[ref10] Elks M. Herbert-Carter J. Smith M. Klement B. Knight B. (2018). Shifting the Curve: Fostering Academic Success in a Diverse Student Body. ACAD MED. 93(1):66–70. 10.1097/acm.0000000000001783 28678099 PMC5753825

[ref11] Ferguson K. J. Wolter E. M. Yarbrough D. B. Carline J. D. Krupat E. (2009). Defining and describing medical learning communities: Results of a national survey. ACAD MED. 84(11):1549–1556. 10.1097/acm.0b013e3181bf5183 19858814

[ref12] Giordani B. Edwards A.S. Segal S.S. Gillum L.H. Lindsay A. (2001). Effectiveness of a formal post-baccalaureate pre-medicine program for underrepresented minority students. ACAD MED. 76(8):844–848. 10.1097/00001888-200108000-00020 11500290

[ref13] Hill L.G. Betz D.L. (2005). Revisiting the Retrospective Pretest. AM J EVAL. 26(4):501–517. 10.1177/1098214005281356

[ref14] Hsieh H. Shannon S. E. (2005). Three approaches to qualitative content analysis. QUAL HEALTH RES. 15(9):1277–1288. 10.1177/1049732305276687 16204405

[ref15] Lipscomb W.D. Mavis B. Fowler L. Vl. Green W.D. Brooks G. L. (2009). The effectiveness of a post baccalaureate program for students from disadvantaged backgrounds. ACAD MED. 84(10):S42–45. 10.1097/acm.0b013e3181b37bd0 19907383

[ref16] Nietfeld J.L. Schraw G. (2002). The effect of knowledge and strategy training on monitoring accuracy. J EDUC RES. 95(3):131–143. 10.1080/00220670209596583

[ref17] Persky A. Dupuis R. (2014). An Eight-year Retrospective Study in “Flipped” Pharmacokinetics Courses. AM J PHARM EDU. 78(10) Article 190. 10.5688/ajpe7810190 PMC4315212 25657377

[ref18] University of New Mexico School of Medicine . hsc.unm.edu/school-of-medicine/education/. AccessedJuly 1, 2017.

[ref19] Sensibaugh C. Madrid N. H-J. Choi H-J. Anderson W. Osgood M. (2017). Undergraduate performance in solving ill-defined biochemistry problems. CBE-Life Sciences Ed. 16(4): ar63. 10.1187/cbe.15-04-0106 PMC5749965 29180350

[ref20] Stalmeijer R.E. (2015). When I say..cognitive apprenticeship. MED EDUC. 49:355–356. 10.1111/medu.12630 25800294

[ref21] White C. Bradley E. Martindale J. Roy P. Patel K. (2014). Why are medical students ‘checking out’ of active learning in a new curriculum? MED EDUC. 48:315–324. 10.1111/medu.12356 24528466

[ref22] Yengo-Kahn A. Baker C. Lomis K. (2017). Medical Students’ Perspectives on Implementing Curriculum Change at One Institution. ACAD MED. 92:455–461. 10.1097/acm.0000000000001569 28099177

[ref23] Zimmerman B.J. Schunk D.H. (2001). Self-regulated learning and academic achievement. Theoretical Perspectives. Lawrence Erlbaum Assoc., Publishers, Mahwah, NJ. 10.4324/9781410601032

