# Supplementary material for: Medical Students at Risk: Practical Lessons after 10 years of a Premedical Enrichment Program
Source: MedEdPublish (2016). 2019 Sep 17;8:175. [Version 1] doi: 10.15694/mep.2019.000175.1 (PMC10911766; doi:10.15694/mep.2019.000175.1)
Supplement: Peer review history

# Reviewer report: Reviewer response for version 1

**DOI:** 10.21956/mep.19877.r30058

**Published:** 2019-11-08

**Authors:** Ken Masters

**Affiliations:** 1 Sultan Qaboos University

This review has been migrated. The reviewer awarded 4 stars out of 5 A truly remarkable and inspiring article dealing with a Premedical Enrichment Program designed to assist medical students at risk. The authors have performed an invaluable study of the program as it existed over a 10-year period, giving clear statistical data and supporting those data with illuminating qualitative data. Mention was made that one of the students was not happy with the program. While the qualitative data quite correctly show illustrative quotations supporting the themes (quite correctly, because that is the majority consensus), the authors do acknowledge in their limitations that there may be positive bias in the reporting. As a result, having even one negative report is important (particularly given such a small sample), and it would have been useful if the authors had explored that a little more, so as to obtain more insight into the problems.Minor issues:• “and importantly, 6) improved” I agree that this point is important, but they all are. So, unless the authors intend to emphasise this point over the others, I would recommend that the “importantly” be removed.• The last portion of the 2nd paragraph in the Methods contains results, and should really have been placed in the Results section.As an aside: the small note that the program is now concluded appears to be a great pity.

Reviewer Expertise:

NA

No decision status is available

# Reviewer report: Reviewer response for version 1

**DOI:** 10.21956/mep.19877.r30057

**Published:** 2019-10-15

**Authors:** Trevor Gibbs

**Affiliations:** 1 AMEE

This review has been migrated. The reviewer awarded 5 stars out of 5 I very much enjoyed reading this well written and important paper covering a very topical subject. I learned much from this paper and enjoyed hearing about the longer term effects of the described programme.I feel that the paper explored several learning theories- social learning and situated learning theory- in their paper - pity that they were not that well referenced. However, this paper is an essential read fro all those involved in student selection, and widening participation activities.

Reviewer Expertise:

NA

No decision status is available
